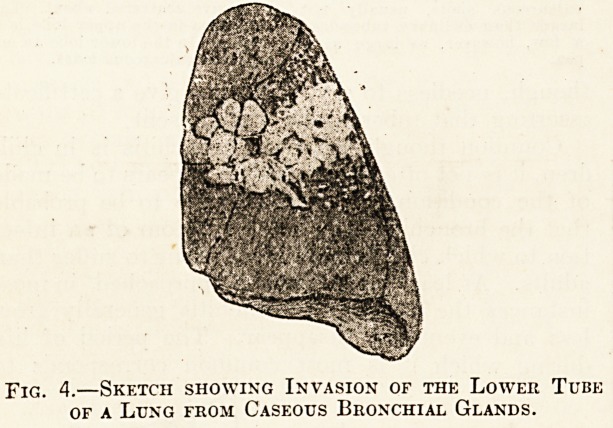# A Few Points Concerning Affections of the Lungs

**Published:** 1911-05-13

**Authors:** Theodore Fisher


### Hospital Clinics.

### I.—A FEW POINTS CONCERNING AFFECTIONS OF THE LUNGS.

By THEODORE FISHER, M.D., F.R.C.P.

Since medical inspection of schools has become general the question of the frequency of chronic tuberculosis of the lungs in children has been a subject upon which very different views have been expressed. Personally, I hold chronic tuberculosis of the lungs in children is extremely rare. Caseation of the mediastinal and bronchial glands is unfortunately a form of tuberculosis which is far from rare in children, and some children in whose chests are hidden such glands may probably—in the loose phraseology sometimes employed—be stated correctly to have "a tendency to consumption." That is to say, at any time tuberculosis of the lungs may possibly arise. This, when it does arise, is generally acute or sub-acute. The majority of so-called "consumptive" children, or children "with

Fig. 1.—The Deformed Chest of Chronic Bronchitis.

a tendency to consumption," are suffering from anæmia and dyspepsia. A considerable number of children, however, are subject to recurrent attacks of bronchitis. These attacks may occur in the summer and in the winter. Occasionally they are most common in the summer, but as a rule the children suffer most in the winter. The children who are the subjects of this form of bronchitis are frequently described by medical men as "consumptive." Now that medical men are visiting the schools we see a considerable number of such children as hospital out-patients whose parents have been advised to take them to a hospital on account of tuberculosis of the lungs.

Quite recently a school medical inspector had been so sure of his diagnosis that arrangements had been made to send a child for some months into the country. All that I was required to do when the child visited the hospital was to fill in the certificate. It so happened that the child had been seen by me from time to time for three or four years on account of recurrent bronchitis. The parents had been somewhat annoyed by the papers sent to them, in consequence of the diagnosis of the school doctor, regarding the danger of infection, and also by the sum they were required to give towards the child's support when away from home. Since every arrangement had been made—even the date of leaving home and the train fixed—it did not come within my province to interfere with what had been done,

Fig 2.

Diffuse fibrosis chiefly of the upper lobe. The fibrosis springs from calcareous spots, usually not larger than ordinary tubercles; a few, however, as large as a pea.

Fibrosis less marked than in the right lung. Calcareous nodules extensive scattered about. The oval area in the upper lobe is a cavity. In the lower lobe an encapsuled calcareous mass.

though, needless to say, I did not give a certificate asserting that tuberculosis was present.

Common though recurrent bronchitis is in children, it is not often that mention appears to be made of the condition. It seems to me to be probable that the bronchitis may be a symptom of an infection to which children are more prone to suffer than adults. At least as puberty is approached in most instances the attacks of bronchitis generally grow less and eventually disappear. The period of life during which it is most common corresponds to

Fig. 3.—A Diagram Showing the Situation of Abscesses of the Lungs in a Boy who Died of Septicæmia, associated with Middle Ear Disease.

that of recurrent vomiting, and during the same period short attacks of fever are not uncommon in which there is neither bronchitis nor vomiting. In all these affections there are two or three days' fever and then rapid convalescence.

It may be of interest to mention in this connection, in order to show how these simple affections may lead to mistakes, that I have met with a boy aged six who since the age of eighteen months was said to have suffered about once a month from attacks of fever lasting about three days. When seen by a medical man during an attack, he was hastened off to a fever hospital, after his return from which place he suffered from scarlet fever. In this boy there was neither bronchitis nor rash. But, curiously enough, I have also met with a case where recurrent bronchitis was accompanied by a rash which was mistaken for scarlet fever. This boy, who when seen was aged eleven years, had for several years suffered from attacks of bronchitis about every six weeks. A rash was accustomed to appear at the time of onset of the bronchitis which—during his stay in a hospital—was said to have been regarded as scarlet fever. Apparently the rash used to be followed by general peeling. When questioned, perhaps with some indication of incredulity, as to the nature of the peeling, it was somewhat emphatically stated "You can see the scales come off him."

Recurrent bronchitis commonly appears quite early in life, and varies considerably in the frequency of the attacks. Some of the children are delicate-looking, and, as already mentioned, are frequently called "consumptive." Others appear to be comparatively healthy. The following are brief notes of cases which happen to be the first two on the list of the index in one of my note-books. They

Fig. 4.—Sketch showing Invasion of the Lower Tube of a Lung from Caseous Bronchial Glands.

are more or less typical. "Evelyn B.——, aged five, one or two attacks of bronchitis before twelve months of age. A bad attack when just over twelve months. Frequent attacks every year since. They are said to be getting more frequent, occurring six or seven a year. The child 'very hot and burning' at onset. Feverish three or four days. Cough very bad for a few days." A later note is more definite regarding the number of attacks, and states that there had been six during the past six months. The next case in my note-book runs as follows: "Madeline C——, aged five. Attacks of bronchitis lasting two or three days occurring every three weeks. Sometimes delirious at night. Seems 'very prostrate' for two or three days and then bright and well until the next attack." Then follows a note as to the dates of the recent attacks. They were, "one in July, one in August, two in September, none in October, one in November."

Most commonly, as soon as the febrile attack is over the cough rapidly disappears, and the child may be in moderately good health or even very good health until the next attack. Occasionally, however, the cough never quite leaves the child. It is customary to speak of chronic bronchitis as rare in children. Possibly this is true, but chronic bronchitis may occur and be a very distressing disease. In some instances it appears to commence as a recurrent bronchitis. After a time the cough, instead of disappearing at the end of an attack, continues. When chronic bronchitis becomes thoroughly established in a child the aspect is one of considerable distress, and the chest may assume the shape of the chest of an adult with chronic bronchitis and emphysema (see fig. 1, which, however, is a sketch taken from a boy who suffered not only from bronchitis but from asthma). A chest of similar shape may be present, however, in marked cases of chronic bronchitis; such, for example, as in a boy aged twelve years, who had suffered from bronchitis since the age of five, after which age there had been several attacks a year. At the age of twelve not only had he a moderately barrel-shaped chest, but presented the curious staring expression which is frequently associated with chronic respiratory distress. It perhaps may be mentioned that the sketch does not represent the condition of deformed chest very accurately. The shoulder should be higher and the chin lower. To return to the boy, he was looked up at his house four years later—that is, when he was sixteen years of age. The attacks of bronchitis were still more or less recurrent, and the information was given, without having been elicited by any question, that they were "worse in the summer than in the winter." However, the cough was said to "never really leave him." In spite of the bronchitis the boy was working at "clicking" in the shoe-trade.

It has been mentioned that the majority of cases of recurrent bronchitis appear completely to recover. In those cases in which there is recurrent bronchitis associated with a chronic cough—that is to say, in cases in which, although the cough is worse at times, there is always some cough, there may be a lesion of the lungs of the nature of dilated bronchial tubes, which accounts for the persistence of the cough. In my experience, however, dilatation of the bronchial tubes generally follows broncho-pneumonia, especially the broncho-pneumonia which follows measles. Whether, however, nearly all cases of chronic cough in children are instances or not of some degree of dilatation of the bronchial tubes, there are many such cases in which evidence of dilatation of the tubes cannot be discovered. While the diagnosis between chronic bronchitis and dilatation of the bronchial tubes may frequently be difficult, it may be interesting to note, in connection with the frequent diagnosis by some observers of chronic tuberculosis in children, that I looked up, a few years after they were first seen, twelve cases of chronic cough. Four of these I had called chronic bronchitis. In one, a boy aged six, afterwards seen at the age of ten, the cough had disappeared for a year, and he had become a healthy-looking boy. In a girl aged five, seen again when nine, the cough still continued. In a boy aged ten (a very bad case) there was some improvement at the age of twelve, and a girl aged twelve had died two years later during an attack of acute bronchitis. In six other cases I had discovered signs either of dilatation of the bronchial tubes or of fibrosis of the lung. All of these were alive at periods of from three to seven years after they were first seen, and the majority had improved in health. Finally, in two cases I had diagnosed tuberculosis. In one of these cases, a girl aged ten, death was found to have taken place, and in the other, although I was unable to see the boy himself, he was said to be in good health. At the age of seven years I had considered a tuberculous cavity to have been present at the apex of the left lung of this boy, but since he was alive and well at the age of eleven I have no doubt that I was mistaken. Dilatation of the bronchial tubes at the apex of a lung I believe to be frequently mistaken for tuberculous disease. It is possible that even in the fatal case in which I had diagnosed tuberculosis that my diagnosis had been wrong and that the disease was chronic fibrosis of the lung. However that may be, it is of interest to note that death had occurred in only one other case, and that a case of chronic bronchitis. Had the chronic cough in other cases indicated the presence of tuberculosis there could hardly fail to have been a larger percentage of deaths.

### Consumption in Children.

My post-mortem experience bears out my clinical experience that chronic tuberculosis of the lungs is rare in children. Autopsies upon cases of chronic dilatation of the bronchial tubes are not uncommon in children, whereas to see a case of chronic phthisis in children in the post-mortem room is very exceptional. Yet even quiescent tuberculosis may occasionally be found. I remember when a student at Guy's seeing a post-mortem examination upon a girl aged about seven or eight years who had died of some acute disease. Towards the apex of one of her lungs was a calcareous nodule with several fibrous bands emanating from it. I have also made a post-mortem examination upon a case of broncho-pneumonia in a boy aged three years who had died of acute broncho-pneumonia, in which an encapsuled caseous mass was found in the upper lobe of the left lung. In a girl, too, who died of tuberculous peritonitis was healed chronic disease of one lung. However, "exceptions prove the rule." The occasional discovery of the presence of chronic or even of healed tuberculosis in the lungs of children only emphasises the infrequency of the discovery. Here it may be remarked that cavities in the lungs of children discovered in the post-mortem room must not be mistaken for chronic cavities. The breaking-down of large masses of caseous broncho-pneumonia may produce a spherical cavity which to the inexperienced may resemble a chronic phthisical cavity. Such a cavity may sometimes be seen in the lung of a child only a few months of age.

More interesting probably than the occasional occurrence of chronic tuberculous disease in the lungs of children are the signs of apparent recovery from acute or sub-acute tuberculosis of the lungs in adult life. Some degree of scarring at the apices of the lungs, as is well known, is an extremely common lesion to be seen in the post-mortem room, but it is perhaps not so generally recognised that scattered calcareous nodules are not very infrequently seen. Curiously enough such nodules appear to be most common in the lower lobes. Whether this presence of calcareous nodules in the lower lobe indicates that the origin of the nodules is not the tubercle bacillus has never been quite clear to me. It is possible that they may represent patches of suppurative broncho-pneumonia from which recovery has taken place. However that may be, from time to time calcareous nodules may be met with scattered throughout both lungs which are undoubtedly of tuberculous nature. For example, in a man who died at the age of twenty-five years of tuberculous meningitis, calcareous nodules in the midst of fibrosis were scattered throughout both lungs. The accompanying diagram (fig. 2), a copy of a figure in my notes, illustrates the condition found. In another case of a middle-aged man who died soon after being discovered in the street in uræmic convulsions each lung was found to contain scattered throughout it from forty to fifty calcareous nodules about the size of large shot, the lower and upper lobes being equally affected. In both of these cases extensive sub-acute tuberculosis of the lungs had evidently at one time been present. As has been mentioned above, the cause of death in one instance was eventually a form of tuberculosis. In spite of the remarkable power that must have been present at one time to resist the tubercle bacillus, the patient succumbed to its attack in another part of the body.

### Why the Apices are Affected.

The reference to the occasional presence of calcareous nodules limited to the lower lobes calls to mind the almost invariable invasion in the first place of the apices of the lungs by tubercle. Recently a physician at a hospital for diseases of the chest mentioned to me as a comparatively new theory that the lodgment of the tubercle bacillus near the apex is due to the fact that it is there the movement of the air is least. A fellow-student of my own, Dr. H. J. Campbell, suggested this many years ago, and I have always been accustomed to tell students that micro-organisms entering the lungs by the air lodge in the position of air stasis or of comparative stasis, and micro-organisms entering by the blood lodge in the position of comparative blood stasis. That is to say, tuberculosis of the lungs generally starts at a point just below the apex, where the opposing movements of the small portion of the lung above the first rib, *i.e.* outside the chest, and movement of the lung below, and inside the chest, meet. At this point the air must be much more still than elsewhere, and here foreign bodies, such as bacilli entering by the air, will rest. When micro-organisms enter the blood, as, for example, in disease of the middle-ear, they will get whirled to the periphery of the blood-stream and lodge in the position of the least blood-movement. This position is generally those edges of the lungs which are comparatively little affected by the movements due to the expansion and contraction of inspiration and expiration. The contiguous borders of the lungs in the axillæ are the most common positions for evidence of the lodging of micro-organisms brought by the blood, and a double row of small abscesses may not uncommonly be seen there. The extreme lower border of the lung may, however, also suffer. The accompanying diagram (fig. 3) taken from a boy aged thirteen, who died of septicæmia associated with disease of the middle-ear, shows abscesses in both of these positions. To return to the subject of the invasion of the lungs by tubercle, as is well known, in children the invasion almost invariably takes place directly from caseous bronchial glands, and the lower lobe is almost as likely to be invaded as the upper. Fig. 4 shows invasion of the lower lobe originating in caseous bronchial glands.

## Figures and Tables

**Fig. 1. f1:**
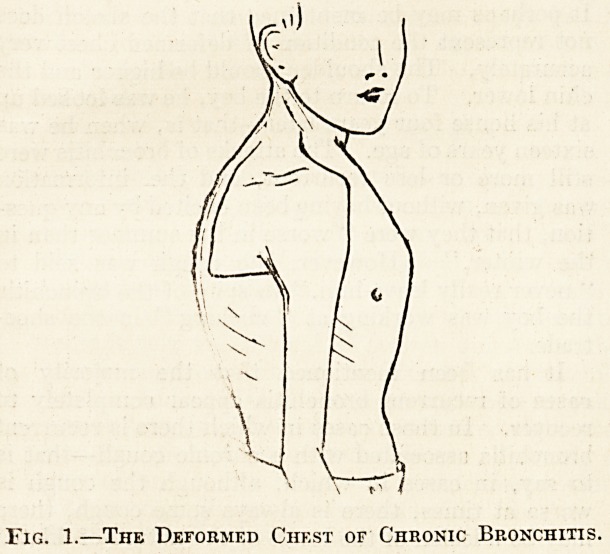


**Fig 2. f2:**
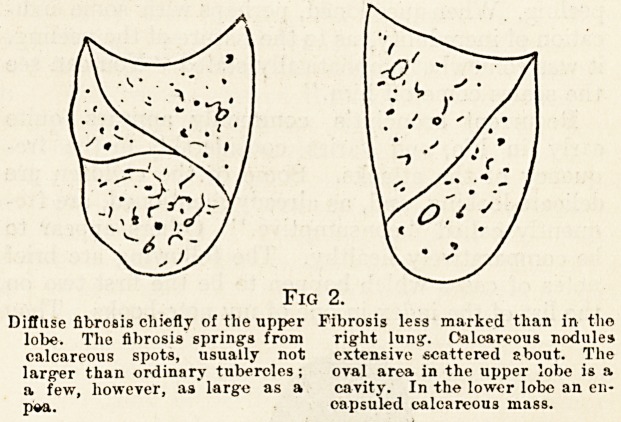


**Fig. 3. f3:**
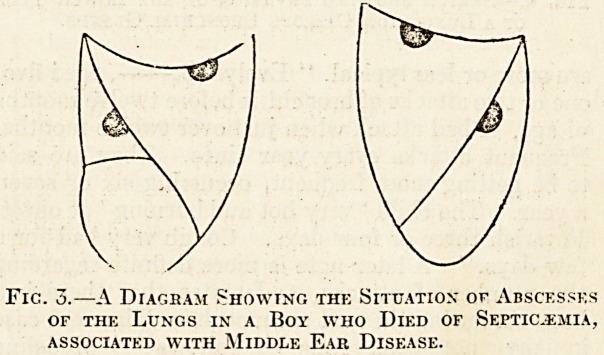


**Fig. 4. f4:**